# Microfluidic Device for the Analysis of Angiogenic Sprouting under Bidirectional Biochemical Gradients

**DOI:** 10.3390/mi11121049

**Published:** 2020-11-27

**Authors:** Keigo Nishimura, Minghao Nie, Shigenori Miura, Shoji Takeuchi

**Affiliations:** 1 Department of Life Sciences, Graduate School of Arts and Sciences, The University of Tokyo, 3-8-1 Komaba, Meguro-ku, Tokyo 153-8902, Japan; nishimura@hybrid.t.u-tokyo.ac.jp; 2 Department of Mechano-Informatics, Graduate School of Information Science and Technology, The University of Tokyo, 7-3-1 Hongo, Bunkyo-ku, Tokyo 113-8656, Japan; nie@hybrid.t.u-tokyo.ac.jp; 3 Institute of Industrial Science (IIS), The University of Tokyo, 4-6-1 Komaba, Meguro-ku, Tokyo 153-8505, Japan; s-miura@iis.u-tokyo.ac.jp; 4 International Research Center for Neurointelligence (WPI-IRCN), The University of Tokyo Institutes for Advanced Study (UTIAS), The University of Tokyo, 7-3-1 Hongo, Bunkyo-ku, Tokyo 113-8656, Japan

**Keywords:** endothelial cells, spheroid culture, trapping, gel scaffold, interstitial flow

## Abstract

In this paper, we developed a spheroid culture device that can trap a spheroid in the trapping site sandwiched by two extracellular matrix gels located at the upper and lower side of the spheroid. This device can form different biochemical gradients by applying target biochemicals separately in upper and lower channels, allowing us to study the angiogenic sprouting under various biochemical gradients in different directions. In the experiments, we confirmed the trapping of the spheroids and demonstrate the investigation on the direction and extent of angiogenic sprouts under unidirectional or bidirectional biochemical gradients. We believe our device can contribute to understanding the pathophysiological phenomena driven by chemical gradients, such as tissue development and tumor angiogenesis.

## 1. Introduction

Angiogenesis is a physiological process in which new blood vessels form from pre-existing blood vessels. The direction of angiogenesis is important both during early embryonic development and in later adulthood; the misdirection of angiogenesis may lead to development failure or loss of vision [1,2]. One of the important factors for determining the direction of angiogenesis is the complex biochemical gradient distribution in the living organism [3]. Various in vitro studies have shown the promotion and modulation of angiogenesis by various pro-angiogenic factors such as vascular endothelial growth factor (VEGF) [4,5,6,7], sphingosine-1-phosphate (S1P) [4,5,8,9,10], and phorbol 12-myristate 13-acetate (PMA) [4,5,11,12]. In addition, cobalt chloride (CoCl_2_) induces a pseudo-hypoxic condition and stimulates the production of VEGF through the upregulation of transcription factor HIF-1α [13]. Recently, the advancement of microfluidic chips has further enabled the application of a biochemical gradient to regulate the direction and morphology of the angiogenic sprouts from a cell monolayer [14,15] or a gel-surrounded spheroid [16,17,18] towards channels with target chemicals. However, the applicable chemical gradients were limited to only one direction; the current systems can only have either a single channel with target chemicals [14,15], or have hydrogel surrounding the spheroids so that most gradients will be bypassed through the gels [16,17,18].

**Figure 1:**
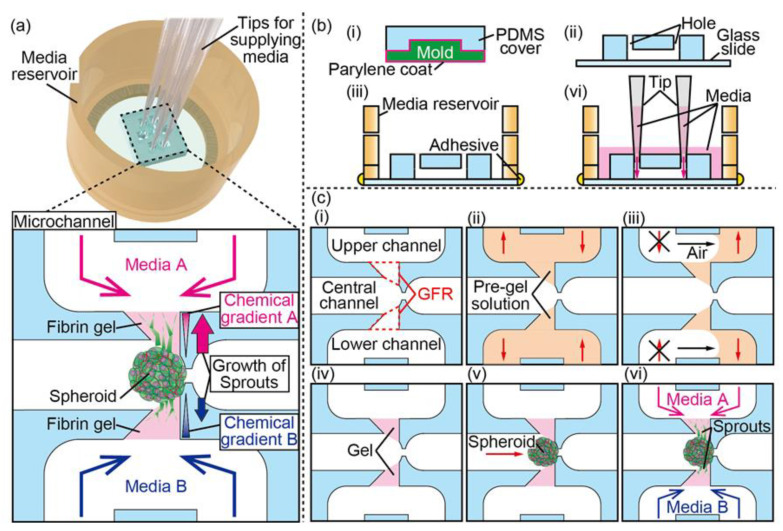
Concept and design of the device and the fabrication process: (**a**) conceptual illustration of the spheroid culture device for bidirectional angiogenic sprouting in chemical gradients; (**b**) the fabrication step of the device and the schematic to form chemical gradients; (**c**) the fabrication steps to form gels and to trap a spheroid in the microchannel of the device.

In this paper, we develop a spheroid culture device that can trap a spheroid in the trapping site sandwiched by two extracellular matrix (ECM) gels located at the upper and lower side of the spheroid. This device can form different biochemical gradients by applying target biochemicals separately in upper and lower channels, allowing us to study the angiogenic sprouting under various biochemical gradients in different directions (Figure 1a). In the experiments, we confirm the trapping of spheroids and the subsequent adherence of the spheroids to the upper and lower ECM gels, which ensure that separate biochemical gradients can be applied to the spheroids. In addition, we demonstrate the investigation on the direction and extent of angiogenic sprouts under unidirectional or bidirectional biochemical gradients.

## 2. Materials and Methods

### 2.1. Materials

Microchannels are fabricated by casting the mixture of polydimethylsiloxane (PDMS) elastomer and a curing agent (Silpot 184 W/C, Dow Corning Toray Co., Ltd., Tokyo, Japan) against a 3D-printed mold. The mold was fabricated with a photocurable resin (HTM140 V2, EnvisionTEC GmbH, Gladbeck, Germany) using a commercial stereolithography machine (Perfactory 4 mini, EnvisionTEC GmbH).

In recent studies, various gels have been developed including natural [19], modified [20], and synthetic hydrogels [21,22,23]. Here, we focused on using natural ECM hydrogels to mimic the natural microenvironments. The pre-gel solution to form the ECM gels consists of 7.2 mg/mL fibrinogen (fibrinogen from bovine plasma type I-S, Sigma-Aldrich Co., LLC, Saint Louis, MO, USA), 0.45 U/mL thrombin (thrombin from bovine plasma, Sigma-Aldrich Co., LLC), 0.29 mg/mL neutralized type-I collagen solution (DME-02, KOKEN Co., Ltd., Tokyo, Japan), 0.15 U/mL aprotinin solution (aprotinin from bovine lung, Sigma-Aldrich Co., LLC) and 36 µM calcium chloride solution (Kanto Chemical Co., Inc., Tokyo, Japan). Each solution was diluted with phosphate-buffered saline (PBS, Cell Science and Technology Institute Inc., Miyagi, Japan) and subsequently sterilized by filtration through a 0.22 μm-pore size membrane filter unit (Millex GV, Durapore^TM^ PVDF bacterial nylon membrane filter, Merck Millipore Ltd., Country Cork, Ireland). When visualizing the ECM gel under a fluorescent microscope, the ECM pre-gel solution was supplemented with 0.04% (*w*/*v*) fluorescent nanobeads (FluoSpheres™ carboxylate-modified microspheres, 0.2 µm, yellow-green fluorescent (505/515), 2% solids, Life Technologies Corp., Carlsbad, CA, USA).

### 2.2. Cell Culture and Spheroid Formation

We cultured normal human pulmonary fibroblasts (HPF, PromoCell GmbH, Heidelberg, Germany) using Dulbecco’s modified Eagle’s media (Nacalai Tesque, Inc., Kyoto, Japan) supplemented with 10% (*v*/*v*) fetal bovine serum (FBS, Thermo Fisher Scientific Inc., Waltham, MA, USA), 1% (*v*/*v*) penicillin/streptomycin and 250 μM l-ascorbic acid phosphate magnesium (Wako Pure Chemical Industries, Ltd., Osaka, Japan). We cultured green fluorescent protein-expressing human umbilical vein endothelial cells (GFP-HUVECs, Angio-Proteomie, Boston, MA, USA) and red fluorescent protein-expressing human umbilical vein endothelial cells (RFP-HUVECs, Angio-Proteomie, USA) in endothelial growth media-2 (EGM-2) media, supplemented with 2% (*v*/*v*) fetal bovine serum (FBS), 0.04% (*v*/*v*) hydrocortisone, 0.4%(*v*/*v*) human fibroblast growth factor-2 (FGF-2), 0.1% (*v*/*v*) vascular endothelial growth factor (VEGF), 0.1% (*v*/*v*) R3-insulin-like growth factor-1 (IGF-1), 0.1% (*v*/*v*) human epidermal growth factor (EGF), 0.1% (*v*/*v*) gentamicin/amphotericin-B, 0.1% (*v*/*v*) heparin (Lonza, Basel, Switzerland) and 1% (*v*/*v*) penicillin/streptomycin (P/S). Since GFP- and RFP-HUVECs are obtained by infecting GFP- or RFP- lentiviral particles according to the manufacturer, these cells stably express GFP and RFP for a long time. To form spheroids, we prepared a cell suspension of HPFs and HUVECs (GFP-HUVECs or RFP-HUVECs) at a ratio of 4: 1 (2.0 × 10^4^ cells: 5.0 × 10^3^ cells per each well) in 200 μL of EGM-2 media. We seeded the cell suspension into a 96-well plate with U-shaped bottom wells (Sumitomo Bakelite Co., Ltd., Tokyo, Japan) and cultured at 37 °C in 5% CO2 atmosphere. Diameters of the spheroids formed were analyzed using Fiji software (https://imagej.net/Fiji).

### 2.3. Device Fabrication

The fabrication of the microfluidic device is illustrated step-by-step in Figure 1b. The proposed device consists of a media reservoir and microchannels. The media reservoir was fabricated with a 3D printer (Agilista-3100, Keyence Corp., Osaka, Japan). The microchannels are fabricated by casting PDMS against a 3D-printed mold. Then, 3D computer-aided design software (Inventor 2018, Autodesk, Inc., San Rafael, CA, USA) was used to design the model of mold. The molds were printed using a stereolithography machine with photocurable resin. To prevent the mold from inhibiting the curing of the PDMS slab caused by under-reacted chemical radicals remnants in the resin, the mold was conformally coated with parylene C (4 μm) using a chemical vapor deposition machine (Parylene Deposition System 2010, Specialty Coating Systems, Inc., Indianapolis, IN, USA). We cast a PDMS-curing agent mixture at a ratio of 10 to 1 (*w*/*w*) on the parylene-coated mold in a 10 cm cell culture dish (Corning Inc., Corning, NY, USA), degassed in a vacuum chamber for 30 min, and cured at 75 °C for 90 min. The formed PDMS slab with the pattern of the microchannels was demolded from the mold and the inlets and outlets were punched with a biopsy punch (tip diameter 1.5 mm, Kai Industries Co., Ltd., Gifu, Japan). To form the microchannels, the surfaces of both the PDMS slab and a round-shaped glass coverslip (30 mm, Matsunami Glass Ind., Ltd., Osaka, Japan) were treated with oxygen plasma (FA-1, Samco, Inc., Kyoto, Japan) and brought together followed by heating at 75 °C for 90 min. The fabricated microchannels were glued on the bottom of the media reservoir with epoxy resin (Araldite, Huntsman Japan Corp., Hyogo, Japan). For visualizing the microchannels, photocurable resin (Norland Optical Adhesive 63, Norland Products Inc., Cranbury, NJ, USA), blue and red ink solution (Pilot Corp., Tokyo, Japan) were introduced into the microchannels.

### 2.4. Gel Formation

Before using the device, we sterilized the device with 70% ethanol and ozone gas with ultraviolet (CoolCLAVE Plus Ozone and UV Personal Sterilizer, Genlantis Inc, San Diego, CA, USA). To form the two pieces of ECM gel, we first pipetted the ECM pre-gel solution into the upper and lower channels using micropipettes. The pipetted pre-gel solution does not leak into the central channel due to surface tension. Then, the solution was removed from the upper and lower channels while only leaving the gels in the designated gel-forming regions (GFR). Then, we incubated the device at 37 °C for more than 1 h to solidify the gel.

### 2.5. Spheroid Trapping

After the gelation, we loaded a spheroid into the central channel by the flow generated with hydrostatic pressure. The hydrostatic pressure was achieved by the increased height of the liquid surface in a pipette tip inserted in the inlet of the central channel. Cross-sectional images of the spheroids were acquired using a confocal laser scanning microscope LSM780 and ZEN imaging software (ZEISS Microscopy, Jena, Germany). To investigate the flow in the central channel driven by various hydrostatic pressures, we introduced 10 μm polystyrene microbeads (PS-R-10.3, microParticles GmbH, Germany) into the pipette tip that was inserted in the islet of the channel. The microbeads were traced with a high-speed microscope (HAS-U2, DITECT Co. Ltd., Tokyo, Japan). Various hydrostatic pressure was achieved by varying the volume of the microbead’s solution at 50, 100, 200 and 300 μL. We tracked the path and analyzed the flow velocity of the microbeads using the Manual Tracking plugin for Fiji software. To trap a spheroid, we inserted a pipet tip in the inlet of the central channel and introduced a spheroid suspended in 300 μL media into the tip. To evaluate the strength of the adhesion of the spheroids to the gels, media were infused in the central channel under constant flow rates using syringe pumps (KDS 210, KD Scientific Inc., Holliston, MA, USA).

### 2.6. Spheroid Culture under Biochemical Gradient

For each of the upper and lower channels, pipette tips were inserted in both the inlet and outlet of the channel. During the media’s change, the media was withdrawn from both the inlet and outlet, then 200 µL of the media was added in only the inlet of each channel. Through the frequent change of the media, which was performed every day, flows induced by hydrostatic pressure were maintained so that the concentration of biochemicals (i.e., the pro-angiogenic factors) in the upper and lower channels could be maintained and stay approximately constant. Additionally, the central channels were connected to the media reservoir and the media reservoir was refurbished with 4 mL media every 2 days.

To culture a spheroid in a device under chemical gradients, we prepared two types of basal media: (i) the VEGF(0) media and (ii) the control media. The VEGF(0) media is endothelial basal media-2 (EBM-2) media, supplemented with 2% (*v*/*v*) FBS and 1% (*v*/*v*) P/S. The control media is EBM-2 media, supplemented with 2% (*v*/*v*) FBS, 0.04% (*v*/*v*) hydrocortisone, 0.4%(*v*/*v*) FGF-2, 0.1% (*v*/*v*) IGF-1, 0.1% (*v*/*v*) EGF, 0.1% (*v*/*v*) gentamicin/amphotericin-B, 0.1% (*v*/*v*) heparin (Lonza, Switzerland) and 1% (*v*/*v*) P/S. To analyze the angiogenic effects of the various concentrations of VEGF, we supplemented 0.01, 0.1, 1, 5, 10, 20 ng/mL VEGF-A165 (human, recombinant, FUJIFILM Wako Pure Chemical Corp., Osaka, Japan) to the VEGF(0) basal media and named the media as the VEGF(0.01) media, the VEGF(0.1) media, the VEGF(1) media, the VEGF(5) media, the VEGF(10) media and the VEGF(20) media. In this experiment, we used media with fewer additives (VEGF(0)) because we wanted to eliminate the impacts of EGM-2 supplements on cell behaviors as much as possible.

To analyze the angiogenic effects of various kinds of chemical gradients, we supplemented each 500 nM S1P, 100 μM CoCl_2_, 50 ng/mL PMA, and 50 and 100 ng/mL VEGF to the control media and named the media as the S1P(+) media, the CoCl_2_(+) media, the PMA(+) media, and the VEGF(50) and VEGF(100) media. In this experiment, we used media with almost the same composition as EGM-2 (control media); here, the aim is to mimic the in vivo environments where there are various factors and see the effects of biochemicals in such complex environments.

### 2.7. Analysis of the Angiogenic Sprouting

To investigate the angiogenic sprouting from spheroids towards the gels, we observed the sprouts and the related cell migration using fluorescence microscopy on day 1, 3, 5, and 7, “day 0” is used to indicate the day of spheroid trapping. We used Fiji software to measure the invasion area of angiogenic sprouts on day 3, 5, and 7 and the length of angiogenic sprouts on day 1, 3, 5, and 7.

## 3. Results and Discussion

### 3.1. Device Design and Fabrication

The proposed device consists of three parallel microchannels (the upper channel, the central channel, and the lower channel; 500 μm in width and 500 μm in height) and two gel-forming regions (GFR), which are located between the central and the upper/lower channels. There is a trapping site between the two GFRs in the central channel, where we can place a spheroid by introducing it into the central channel (Figure 1b); this trapping structure was modified from the one in the previous reports [24,25]. Since the formed gels function as scaffolds for cells, the trapped spheroid can attach to the gels and form angiogenic sprouts in the gels by culturing the spheroids. By introducing the different biochemicals in each channel, we can form different chemical gradients in two different directions (Figure 1c).

**Figure 2:**
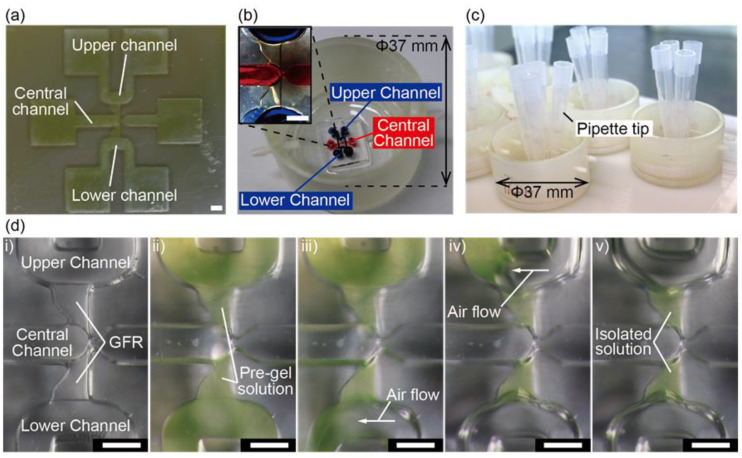
Fabrication of the device and the formation of gels: (**a**) 3D-printed micromold for the microchannel; (**b**) the entire device colored with inks and the enlarged view of the microchannel; (**c**) photograph of the device during the culture under chemical gradients; (**d**) introduction and isolation of pre-gel solutions containing fluorescent nanobeads in gel-forming regions (GFRs). Scale bars are 500 μm.

Figure 2b shows the fabricated device, where the microchannels are colored with blue and red inks. The spheroids were then cultured with four pipet tips inserted in every inlet of the upper and the lower channels (Figure 2c). The dimensions of the microchannel are shown in Figure S1.

The gel consists of fibrin and collagen to facilitate angiogenic sprouting. Pre-gel solutions introduced in the upper, lower channels did not leak into the central channel. By manually sucking the pre-gel solution from the inlets of the upper and the lower channels, we successfully isolated the pre-gel solution in the GFRs (Figure 2d). This isolation was achieved due to the difference of the heights of the upper, lower, and central channels (500 μm) and the GFRs (400 μm). After gelation, the majority of the streamlines of the flow are mainly in the channels, not in the GFRs, showing the detention of the gels in the GFRs.

### 3.2. Spheroid Trapping

**Figure 3:**
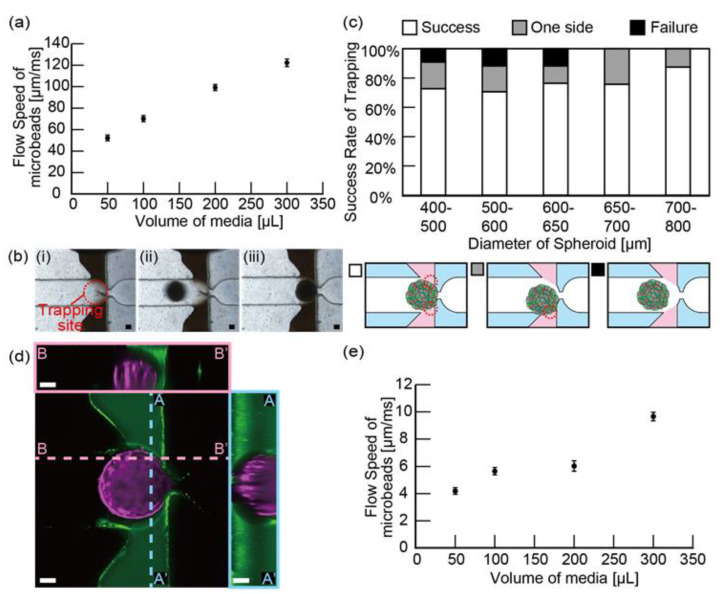
Evaluation of the trapping system of the culture device: (**a**) graph of the flow speed of microbeads in the central channel against the volume of media loaded in the tip before trapping a spheroid. The results are shown as the mean ± standard error (s.e.) of four devices; (**b**) sequential photograph of the trapping system. The spheroid consists of human pulmonary fibroblasts (HPFs) and red fluorescent protein-expressing human umbilical vein endothelial cells (RFP-HUVECs); (**c**) graph of the flow speed of microbeads in the central channel against the volume of media loaded in the tip after trapping a spheroid. The results are shown as the mean ± standard error (s.e.) of four devices; (**d**) graph of success rates of spheroid trapping in each diameter of spheroid. The value was counted from devices where the diameter of spheroid was 400–500 μm (*n* = 11), 500–600 μm (*n* = 17), 600–650 μm (*n* = 17), 650–700 μm (*n* = 33), and 700–800 μm (*n* = 8); (**e**) confocal microscopic image of a gel stained with green fluorescent nanobeads (green) and a spheroid, with x-z and y-z cross-sectional views. Scale bars are 100 μm.

Before trapping the spheroids, we first observed the media flow in the central channel caused by the hydrostatic pressure; the hydrostatic pressure of media was generated by inserting the pipette tip in the inlet of the central channel and increasing the height of the media in the tip. To visualize the flow, we infused microbeads’ suspension media through a pipette tip inserted into the inlet of the central channel. As a result, the flow rate of microbeads increased linearly as the volume of microbeads’ suspension media increased (Figure 3a). We also tracked the behavior of the microbeads and found that a converging streamline was formed at the narrowed trapping site (Video S1). These results suggest that the flow of media can be used to trap spheroids in the center of the channel and promote the contact of spheroids with both sides of the gels.

Next, we successfully trapped the spheroids (HPFs and HUVECs at a ratio of 4:1) by adding a spheroid (400–800 μm in diameter) in the pipette tip with 300 μL media; this trapping was achieved thanks to a narrowed channel (200 μm in width and 400 μm in height) in the trapping site. Figure 3b shows that a spheroid introduced in the central channel was trapped in the trapping site.

To assess the efficiency of spheroid trapping, we measured the percentage of successful trapping according to the size of the spheroids. As a result, the success rates of spheroid trapping were 73% (diameter(dia.) = 400–500 μm; 18% unilateral only, 9% bilateral failure, *n* = 11), 71% (dia = 500–600 μm; 18% unilateral only, 12% bilateral failure, *n* = 17), 76% (dia = 600–650 μm; 12% unilateral failure, 12% bilateral failure, *n* = 17), 76% (dia = 650–700 μm; 24% unilateral failure, 0% bilateral failure, *n* = 33), and 88% (dia = 700–800 μm; 13% unilateral failure, 0% bilateral failure, *n* = 8) (Figure 3c). A slight difference in the successful ratio is because the flow of the media during the spheroid trapping anchors the spheroid to the trapping site and supports adhesion to the gels, even when the size of the spheroid is different. Regarding the spheroid larger than 650 μm, the channel (500 μm in height and width) deforms the shape of the spheroids to fit the shape of the channel and allows for tight adhesion to the gels.

To visualize the adhesion of spheroids to gels, we added green fluorescent nanobeads in the gels and trapped a spheroid containing red fluorescent protein-expressing HUVECs. The cross-sectional images taken by a confocal microscope showed that the spheroid was placed in the vicinity of the gel (Figure 3d). This result suggests that our device facilitates the trapped spheroid to attach to the gels during the overnight culture of the spheroid. After the overnight culture of the trapped spheroid, we infused media inversely from the outlet of the central channel to confirm the adhesion of the spheroid to gels. As a result, the spheroid was anchored to gels until the flow rate was under 3800 µL/m (Video S2). This result indicates that the trapped spheroid firmly adheres to gels by culturing overnight.

After the trapping of the spheroid, we introduced microbead-suspended media through a pipette tip to analyze the flow properties affected by the trapped spheroids. As a result, with the increasing volume of microbead-suspended media, the flow rate of microbeads increased, but the increase was less linear (Figure 3e), indicating that the spheroids fitted into the trapping site cause difficulties for the media to flow through the central channel. During the experiment, the spheroid was not squeezed through the trapping site, nor did the gels break. In addition, when we followed the behavior of microbeads, we observed that microbeads flowed in the center of the channel, changed direction to the outer edge of the channel to pass through the periphery of the spheroid, and then changed direction to the center of the channel again to flow through the narrowed trapping site (Video S3). This result suggests that even after spheroids are captured in the central channel, the slight forward flow of media anchors the spheroids to the central position and facilitates the attachment to the gel during culturing overnight.

### 3.3. Analysis of the Angiogenic Sprouting in Media with Various Concentrations of VEGF

**Figure 4:**
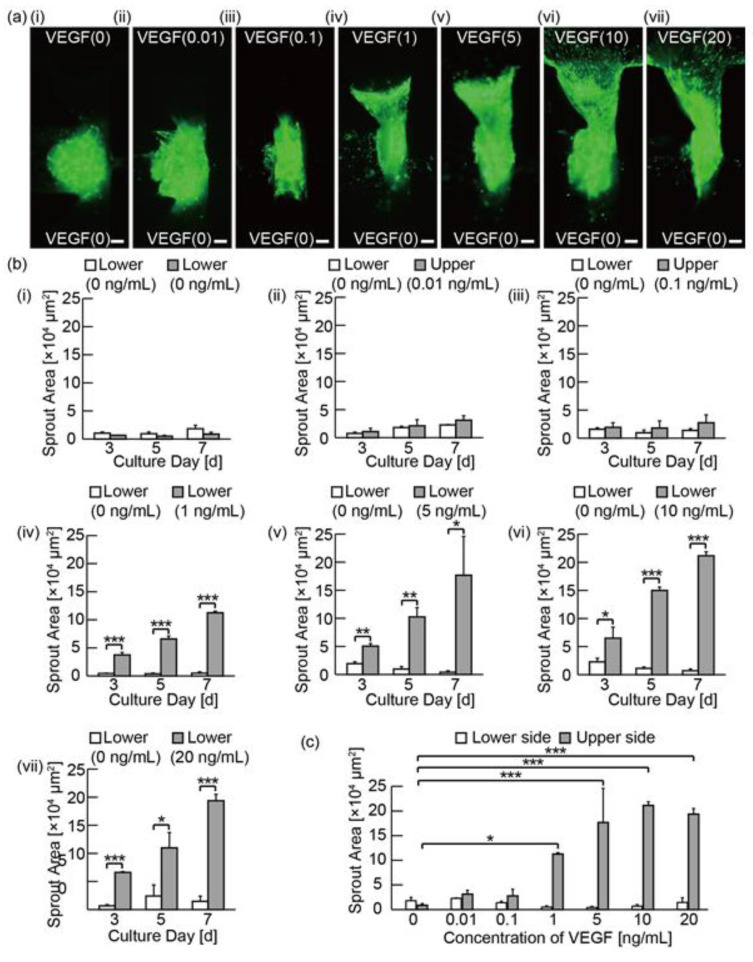
Evaluation of bidirectional angiogenic sprouts induced with vascular endothelial growth factor (VEGF)-containing media: (**a**) fluorescent microscopies of cultured spheroids composed of HPFs and GFP-HUVECs under chemical gradients with different concentration of VEGF-A165 (0, 0.01, 0.1, 1, 5, 10, and 20 ng/mL) on day 7; (**b**) Graphs of invasion area of angiogenic sprouts during the culture underc VEGF gradients on day 3, 5, and 7. The results are shown as the mean ± standard error (s.e.) of 3–5 devices (*n* = 4 (b-i, b-iv, b-vi), *n* = 3 (b-ii, b-v), *n* = 5 (b-iii, b-vii)). * *p* < 0.05, ** *p* < 0.01, *** *p* < 0.001 (Student’s *t*-test); (**c**) Summary graph of invasion area of angiogenic sprouts induced in media with various concentration of VEGF on day 7. * *p* < 0.05, *** *p* < 0.001 (Dunnett’s test). Scale bars are 100 µm.

To investigate the effect of VEGF on angiogenic sprouts, spheroids were cultured for 7 days under the chemical gradients of EBM-2 media supplemented with FBS and P/S (the VEGF(0) media) from the lower channel and the media with various concentrations of VEGF-A165 added to the VEGF(0) media from the upper channel. In detail, the supplemented concentrations are 0.01 ng/mL, 0.1 ng/mL, 1 ng/mL, 5 ng/mL, 10 ng/mL, and 20 ng/mL, the supplemented media are named the VEGF(0.01) media, the VEGF(0.1) media, the VEGF(1) media, the VEGF(5) media, the VEGF(10) media, and the VEGF(20) media, respectively. Figure 4a shows the distribution of GFP-HUVECs on day 7. Figure 4b shows the invasion area of angiogenic sprouts on day 3, 5, and 7. As a result, when the range of VEGF concentrations is 0–0.1 ng/mL, the absolute invasion area of angiogenic sprouts in the direction of the upper channel is small. The difference in the invasion area of the angiogenic sprouts between the directions of the upper and the lower channel is also small; there were no significant differences statistically. In contrast, when the range of VEGF concentrations is 1–20 ng/mL, there were statistically significant differences in the invasion area of angiogenic sprouts between the direction of the upper and the lower channels on day 3, 5, and 7. Additionally, for the conditions of VEGF(1), VEGF(5), VEGF(10), and VEGF(20), the differences in the invasion area in the upper direction had significant differences compared to the condition of VEGF(0) (Figure 4c). These results indicate that VEGF promotes GFP-HUCVECs to sprout towards the upper channel. As the lengths of angiogenic sprouts in GFRs were measured, we found a similar trend to that of the invasion area measurements (Figure S2). In general, VEGF receptors are known to be activated and cause the release of the proteinases under the stimulations of VEGF and other various biochemical factors [26]. Although we have not confirmed the activation of the VEGF receptors and the release of proteases, our results on the formation of angiogenic sprouts and the migration of HUVECs can indirectly indicate the activation of VEGF receptors and the release of the proteinases.

### 3.4. Analysis of Angiogenic Sprouts in Media with Various Kind of Biochemicals

**Figure 5:**
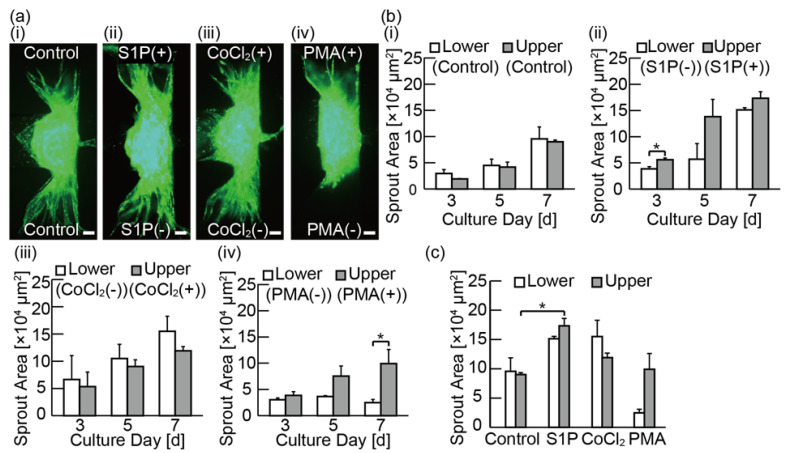
Evaluation of bidirectional angiogenic sprouts induced by media with various pro-angiogenic factors and biochemicals: (**a**) fluorescent microscopies of cultured spheroids composed of HPFs and green fluorescent protein-expressing human umbilical vein endothelial cells (GFP-HUVECs) under various chemical gradients on day 7. The biochemicals used in this experiment are 500 nM sphingosine-1-phosphate (S1P), 100 μM cobalt chloride (CoCl_2_), and 50-ng/mL phorbol 12-myristate 13-acetate (PMA) added in the control media (endothelial basal media-2 (EBM-2) media supplemented with fetal bovine serum (FBS), hydrocortisone, human fibroblast growth factor-2 (FGF-2), R3-insulin-like growth factor-1 (IGF-1), human epidermal growth factor (EGF), gentamicin/amphotericin-B, heparin and penicillin/streptomycin (P/S); (**b**) graphs of invasion area of angiogenic sprouts during the culture under various chemical gradients on day 3, 5, and 7. The results are shown as the mean ± standard error (s.e.) of 3 devices. * *p* < 0.05, (Student’s *t*-test); (**c**) summary graph of invasion area of angiogenic sprouts induced in media with various biochemicals on day 7. * *p* < 0.05 (Dunnett’s test). Scale bars are 100 µm.

To investigate the effects of various pro-angiogenic factors, spheroids were cultured for 7 days under the biochemical gradients generated with the control media (EBM-2 media supplemented with FBS, hydrocortisone, FGF-2, IGF-1, EGF, gentamicin/amphotericin-B, heparin, and P/S) from the lower channel and the control media containing various pro-angiogenic factors and biochemicals (500 nM S1P, 100 μM CoCl_2_, 50 ng/mL PMA, and 50 and 100 ng/mL VEGF-A165) from the upper channel. Figure 5a shows the images of GFP-HUVECs on day 7. Figure 5b shows the invasion area of angiogenic sprouts on days 3, 5, and 7 under each biochemical gradient condition. The length of angiogenic sprouts was also measured (Figure S3). The results showed that under the condition using the control media on both sides, angiogenic sprouts were induced towards both the upper and the lower channels; this result is due to the angiogenesis-promoting effects of FGF-2 IGF-1, EGF in the control media [27,28].

Under the condition of S1P(+) media, the invasion area was enlarged significantly compared to the result under the control media (Figure 5c). Interestingly, despite the fact that the S1P(+) media was applied from the upper channel, the invasion areas of angiogenic sprouts in both the upper and the lower channels have no statistically significant differences on day 7. With the gradient of CoCl_2_, there was also a slight tendency of the invasion area to increase in both directions as compared to that of the condition using the control media on both channels (Figure 5c). Under the condition of PMA media, there was a statistically significant difference between the invasion area of the upper channel and that of the lower channel on day 7 (Figure 5b-iv), indicating the chemotactic effects of the PMA. These results demonstrate that our device is applicable to distinguish chemotactic and chemokinetic effects of several pro-angiogenic factors and biochemicals on angiogenic sprouts.

**Figure 6:**
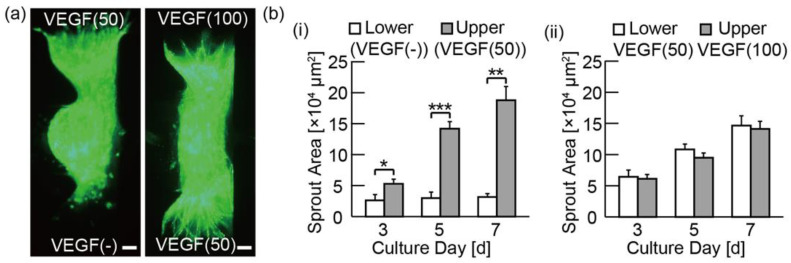
Evaluation of bidirectional angiogenic sprouts induced by media with VEGF: (**a**) fluorescent microscopies of cultured spheroids composed of HPFs and GFP-HUVECs under VEGF-contained media on day 7; (**b**) graphs of invasion area of angiogenic sprouts during the culture under VEGF on day 3, 5, and 7. The results are shown as the mean ± standard error (s.e.) of 3 devices. * *p* < 0.05, ** *p* < 0.01, *** *p* < 0.001 (Student’s *t*-test). Scale bars are 100 µm.

Finally, using VEGF, we compared the effects of unidirectional and bidirectional chemical gradients by culturing a spheroid under the two different conditions: (i) using the control media from the lower channel and the VEGF(50) media from the upper channel, (ii) using the VEGF(50) media from the lower channel and the VEGF(100) media from the upper channel. As a result of condition (i), we observed the asymmetric angiogenic sprouts (Figure 6a) compared to the result using a control media shown in Figure 5a; there was also a statistically significant difference between the invasion area in the upper channel and the lower channel (Figure 6b). These results clearly show the chemotactic effect of VEGF even in the control media that contained other pro-angiogenic factors. As a result of condition (ii), angiogenic sprouts were formed towards both the upper and the lower channels; in this condition, there was no statistically significant difference between the invasion area of the upper and the lower channels. This result indicates that the chemotactic effect of VEGF is positive on both sides of a spheroid, suggesting that our device achieves the application of bidirectional chemical gradients in both the upper and the lower channels.

## 4. Conclusions

In this study, we proposed a device for trapping and culturing a spheroid sandwiched by two gels located on the upper side and the lower side of the spheroid. This setup enabled the analysis of angiogenic sprouts under bidirectional chemical gradients. Using our device, we found that the direction and the length of angiogenic sprouts were affected depending on the type and the gradient directions of pro-angiogenic factors and biochemicals. The advantages of our method are as follows: (i) the high efficiency of spheroid trapping, (ii) the assessment of the angiogenic sprouts not only by the invasion area and length but also by the directionality, and (iii) the formation of bidirectional gradients of biochemicals. Therefore, we believe that our device is applicable to analyze the effects of chemical gradients on cell behaviors, in multi-directions, which is important for understanding the pathophysiological phenomena driven by chemical gradients, such as tissue development and tumor angiogenesis.

## Supplementary Materials

The following are available online at https://www.mdpi.com/2072-666X/11/12/1049/s1, Figure S1: Design of the microchannel of the device, Figure S2: Evaluation of bidirectional angiogenic sprouting induced with VEGF-containing media, Figure S3: Evaluation of bidirectional angiogenic sprouting induced with media containing various chemical stimulators of angiogenic sprouting, Video S1: Analysis of the flow using microbeads without trapped spheroids (Media volume: 50 µL, Speed: ×0.053), Video S2: Evaluation of the strength of the adhesion of a trapped spheroid to gels (Flow rate: 3800 µL/min, Speed: ×40), Video S3: Analysis of the flow using microbeads with a trapped spheroid (Media volume: 200 µL, Speed: ×0.067).
