# Supplementary material for: Microfluidic Device for the Analysis of Angiogenic Sprouting under Bidirectional Biochemical Gradients
Source: Micromachines (Basel). 2020 Nov 27;11(12):1049. doi: 10.3390/mi11121049 (PMC7761305; doi:10.3390/mi11121049)
Supplement: Supplementary file 1 [file micromachines-11-01049-s001.zip › K. Nishimura, et al._Supplementary Materials/K. Nishimura, et al._Supplementary Materials.pdf]

Supplementary Materials: Microfluidic device for the analysis of angiogenic sprouting under bidirectional biochemical gradients

Keigo Nishimura, Minghao Nie, Shigenori Miura and Shoji Takeuchi *

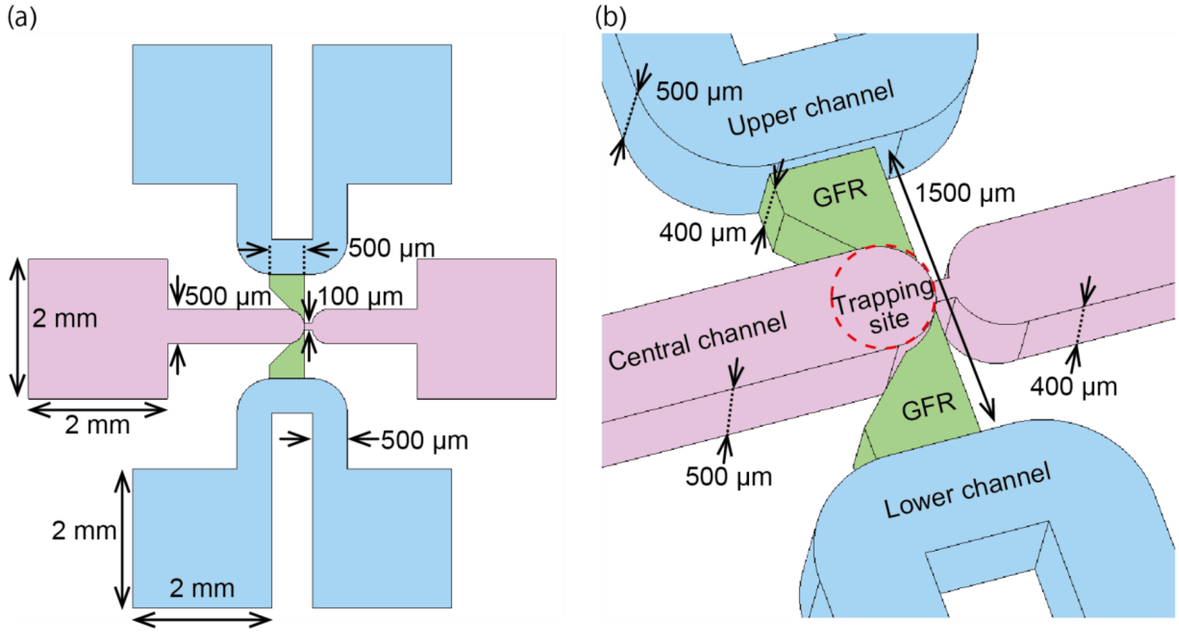

Figure S1. Design of the microchannel of the device: (a) top view of the design; (b) perspective view of the design.

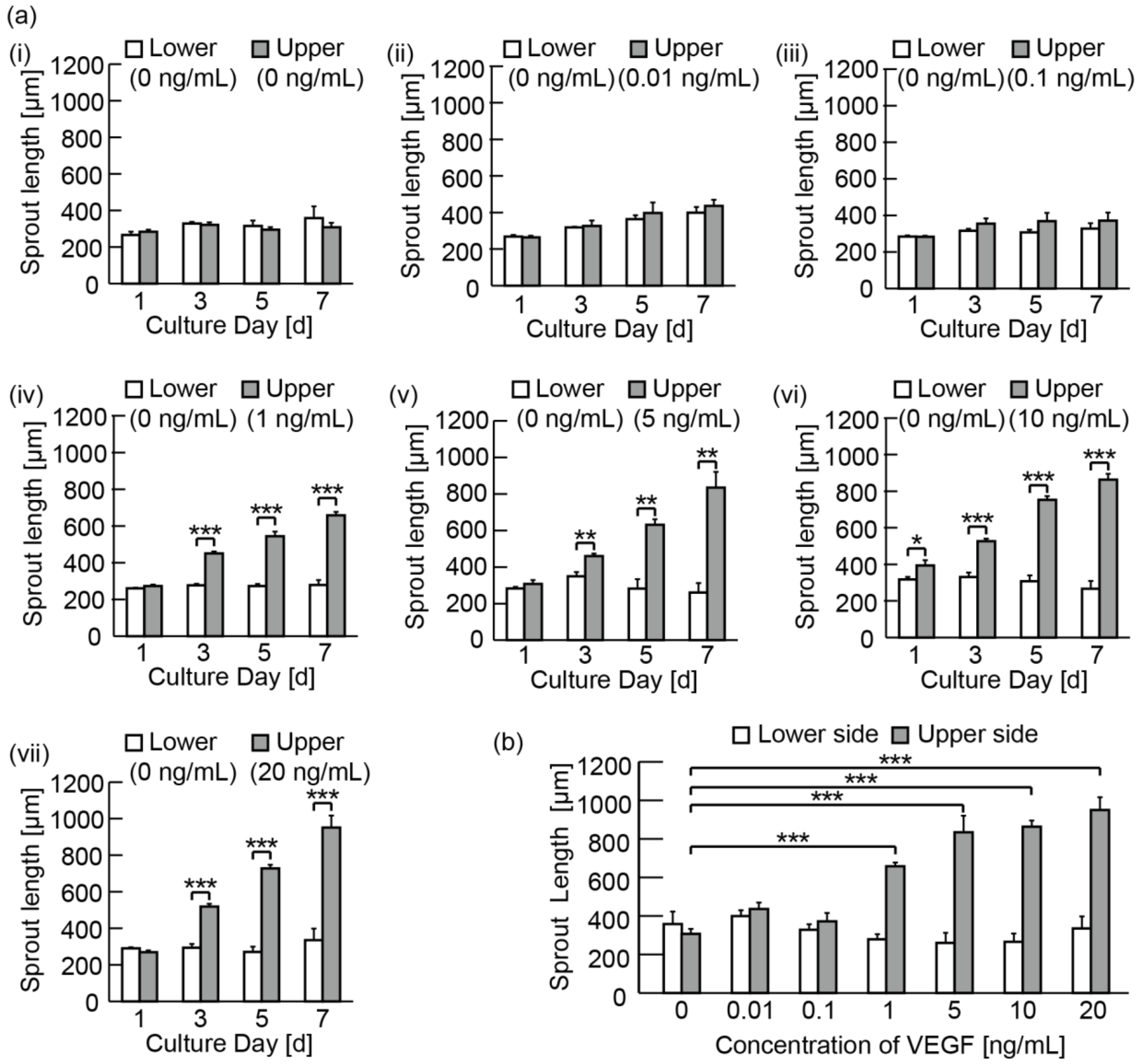

Figure S2. Evaluation of bidirectional angiogenic sprouting induced with VEGF-containing media: (a) graphs of angiogenic sprout length during the culture in VEGF gradients on day 1, 3, 5, and 7. The results are shown as the mean ± standard error (s.e.) of 3–5 devices (*n* = 4 (a-i, a-iv, a-vi), *n* = 3 (a-ii, a-v), *n* = 5 (a-iii, a-vii)). *: p < 0.05, **: p < 0.01, ***: p < 0.001 (Student's t-test); (b) Summary graph of angiogenic sprout length induced in media with various concentration of VEGF on day 7. ***: p < 0.001 (Dunnett's test).

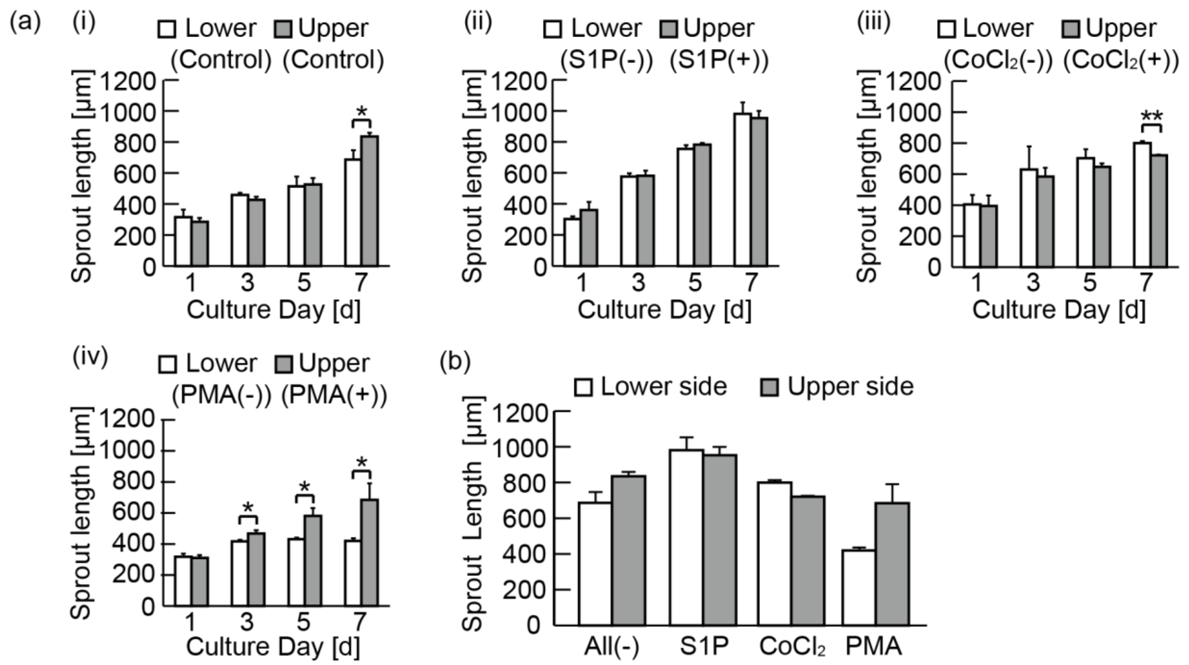

Figure S3. Evaluation of bidirectional angiogenic sprouting induced with media containing various chemical stimulators of angiogenic sprouting: (a) graphs of length of angiogenic sprouts during the culture under various chemical gradients on day 1, 3, 5, and 7. The results are shown as the mean ± standard error (s.e.) of 3 devices. *: $p < 0.05$, **: $p < 0.01$ (Student's t-test); (b) summary graph of length of angiogenic sprouts induced in media with various chemicals on day 7.

Supplementary Video 1. Analysis of the flow using microbeads without trapped spheroids

(Media volume: 50 μL, Speed: ×0.053)

Supplementary Video 2. Evaluation on the strength of the adhesion of a trapped spheroid to gels

(Flow rate: 3800 μL/min, Speed: ×40)

Supplementary Video 3. Analysis of the flow using microbeads with a trapped spheroid

(Media volume: 200 μL, Speed: ×0.067)
